# Cigarette smoking and smokeless tobacco use among male south Asian migrants in the United Arab Emirates: a cross-sectional study

**DOI:** 10.1186/s12889-020-08942-9

**Published:** 2020-05-30

**Authors:** Raghib Ali, Tom Loney, Mohammed Al-Houqani, Iain Blair, Faisal Aziz, Salma Al Dhaheri, Iffat El Barazi, Elpidoforos S. Soteriades, Syed M. Shah

**Affiliations:** 1 Public Health Research Center, New York University Abu Dhabi, Abu Dhabi, United Arab Emirates; 2 College of Medicine and Health Sciences, Institute of Public Health, United Arab Emirates University, PO Box 17666, Al Ain, United Arab Emirates; 3 College of Medicine, Mohammed Bin Rashid University of Medicine and Health Sciences, Dubai, United Arab Emirates; 4 College of Medicine and Health Sciences, Department of Internal Medicine, United Arab Emirates University, Al Aim, United Arab Emirates; 5 Department of Endocrinology and Diabetology, Medical University Graz, Graz, Austria; 6 Health Promotion, Department of Health, Abu Dhabi, United Arab Emirates; 7 Department of Environmental Health, Environmental and Occupational Medicine and Epidemiology (EOME), Harvard T. H Chan School of Public Health, Boston, MA USA; 8 Department of Family Medicine, Aga Khan University, Karachi, Pakistan; 9 Zayed Centre for Health Sciences, UAE University, Al Ain, United Arab Emirates

**Keywords:** Cigarette smoking, Smokeless tobacco use, Prevalence, Migrants, South Asian, United Arab Emirates

## Abstract

**Background:**

Few data were available on smoking and smokeless tobacco use in South Asian migrants in the United Arab Emirates (UAE). This study aimed to identify the prevalence and correlates of cigarette smoking and smokeless tobacco use in male South Asian migrants in the UAE.

**Methods:**

We used a cross-sectional study to recruit a random representative sample of male South Asian migrants, including Indian (*n* = 433), Pakistani (*n* = 383) and Bangladeshi (*n* = 559) nationalities. We used multivariable logistic regression analysis to identify significant correlates of cigarettes smoking and smokeless tobacco use.

**Results:**

1375 South Asian migrant adult males participated in the study (response rate 76%) with a mean age of 34 years (SD ± 10). The overall prevalence of cigarette smoking was 28% (95%CI 25–30%) and smokeless tobacco use was 11% (95%CI 10–13%). The prevalence of current cigarette smoking was 21, 23, and 37% among participants from India, Pakistan and Bangladesh, respectively. The prevalence of current smokeless tobacco use was 6, 12, and 16% for Indian, Pakistani, and Bangladeshi participants, respectively. Among study participants, Bangladeshi nationality, hypertension, and alcohol use were significant correlates of current cigarette smoking. Significant correlates of smokeless tobacco use included increased age, less than college level education, alcohol use, and Pakistani or Bangladeshi nationality.

**Conclusions:**

Current smoking and smokeless tobacco use in South Asian migrants represent a significant public health burden in the UAE. Effective public health measures are needed to reduce tobacco use in this migrant population.

## Background

Tobacco use is the single most preventable cause of death in the world today leading to over 6 million fatalities each year [1]. More than 5 million of those deaths are the result of direct tobacco use and the number of deaths will increase to 10 million per year by the year 2030 if appropriate and improved preventive measures are not implemented [1]. Moreover, 70% of these projected fatalities are expected to occur in low- and middle-income countries [1].

Tobacco use is a major risk factor for cardiovascular disease (CVD), cancer, and a wide range of chronic diseases especially chronic respiratory diseases [2, 3]. Manufactured cigarettes are the most common type of tobacco consumed worldwide but other forms of tobacco, such as chewing tobacco including snuff use in South Asia and shisha smoking (water pipe) in the Middle East, are also quite common in these particular regions [4, 5]. Smokeless tobacco (SLT) is also a major risk factor for oropharyngeal cancer as well as an increased risk of CVD [6, 7].

South Asians, estimated at 25% of the world’s population, represent one of the highest proportions of the global population, affected by current and future disease burden due to tobacco consumption. A high prevalence of tobacco use in this region has been reported, with 40% of males and 5% of females in India, 45% of males and 6% of females in Pakistan, and an overall tobacco use prevalence of 21%. Bangladesh has one of the highest reported prevalence of tobacco use with 58% among males and 29% in females, with an overall tobacco use of 43% [8]. Studies of migrant populations in economically developed countries show that higher tobacco use, and exposure to other environmental factors place migrants at a higher risk for CVD and other health problems [9, 10].

Cigarette smoking is also associated with a significant loss of work productivity. Prospective cohort studies have shown that compared with non-smokers, current smokers had a 33% increase in the risk of absenteeism and were absent for an average of 2.7 more days per year compared to non-smokers [11]. The total cost of absenteeism due to smoking was estimated to be £1.4 billion in UK in one year. In contrast, studies in China, the European Union, and the United States (US), have shown that quitting smoking increased work productivity [12].

The United Arab Emirates (UAE) has one of the largest proportions of a culturally diverse migrant population. Expatriate workers account for almost 80% of the UAE’s total population and about two-thirds are South Asian migrant workers from India, Pakistan, and Bangladesh [13]. Research on the prevalence of tobacco use among UAE nationals (Emiratis) has previously been reported as 24.3% in males and 0.8% in females [14]; however, there have been no studies on tobacco use among South Asian migrants in the UAE or any Gulf countries.

This study aims to estimate the prevalence and correlates of cigarette smoking and smokeless tobacco use among South Asian migrants in the UAE who originated from India, Pakistan, and Bangladesh.

## Methods

### Study design and ethics

The study used a cross-sectional design. Ethical approval was obtained from the Al Ain Medical District Human Research Ethics Committee (AAMDHREC 10/21), and the Abu Dhabi Health Services Company’s Research Committee (08 February 2012). Study participants signed a written informed consent.

### Selection of study participants

The target study population consisted of all male South Asian migrant workers aged 18 years and older from India, Pakistan, and Bangladesh. We used the formula for binomial distribution [n = ^z^zα^2^ p (1- p)/d^2^] to estimate the sample size for our study, where n is the sample size; zα is the normal deviate (1.96) at 5% level of significance. The estimated prevalence (P) of cigarette smoking (estimated at ~ 50% as data on the prevalence of cigarette smoking and use of smokeless tobacco amongst South Asians in the UAE were not available), the precision of estimate (5%), and decided to recruit 1800 participants.

The Abu Dhabi Health Services Company or SEHA (the Arabic word for Health) provides health care in the Emirate of Abu Dhabi, through hospitals, clinics, and ambulatory health care centers. Ten of the ambulatory health care centers are disease prevention and screening centers, primarily for infectious disease screening required for residency visas. All expatriate workers seeking employment or renewing their visa in the UAE are required by law to undergo health and communicable disease screening at one of the screening centers. The sampling frame in our study was a list of all expatriate workers from India, Pakistan, and Bangladesh who were enrolled for medical examination at the only visa screening center in the city of Al Ain, Abu Dhabi during the process of obtaining a new or renewing their visa in 2012 [15–17].

### Inclusion criteria

The sampling frame included male adult (age ≥ 18 years) South Asian migrant workers from India, Pakistan and Bangladesh, attending visa screening in Al Ain, Abu Dhabi. Briefly, study participants had to self-identify with a South Asian ethnicity of India, Pakistan, or Bangladesh, be an adult aged ≥18 years, and be able to speak and /or read Urdu, Hindi or Bengali, and had to provide a written informed consent to be eligible to participate in the study. Due to the low literacy rate among the South Asian expatriate population in the UAE, the study questionnaire was interviewer-administered and all interviews were conducted in Urdu, Hindi, or Bengali, led by a native Urdu, Hindi, or Bengali speaking research assistant who had received appropriate training. Every third person on the list of eligible participants registered for the visa screening was invited to participant in the study and it took six months to recruit the study sample.

### Measures

We used an adapted version of the World Health Organization (WHO) global standard “STEPS” survey questionnaire titled “Chronic Diseases Prevention in Immigrants: putting CVD risk factors on surveillance screen” for population-based assessment of the prevalence of noncommunicable diseases (NCDs) risk factors including cigarette smoking and use of smokeless tobacco [18]. Moreover, we adapted the WHO STEPS survey questionnaire to include South Asian tobacco product questions such as ever or current use of any smokeless tobacco such as tobacco chew or snuff, masher, zarda, bidis, gutka, mawa, paan masala betel nut and supari with tobacco, as indicators of smokeless tobacco use. A study in South Asians has shown that inclusion of such questions improves the estimation of the prevalence of smokeless tobacco use (21.2%) as compared to a global standard questionnaire (4.3%) [19]. Study participants were classified as current smokers if they answered yes to the question, *“*have you ever smoked cigarettes, cigars /biddies/or shisha?”

We used an interviewer-administered survey because of the low literacy in South Asian migrants in the UAE. Studies have shown a higher sensitivity (5.2%) with interviewer administered self-reports of tobacco smoking than that of self-administered survey reports, but this difference was not significantly different from zero (*p* = 0.135) [20].

We collected information on demographics lifestyle factors, family and personal disease history, home country residence setting (rural, urban, semi-urban), occupation, monthly salary (UAE dirhams, AED; USD1.00 ~ AED3.67), years lived in the UAE, current type of accommodation, history of current and past cigarette smoking and tobacco chewing, history of exposure to second-hand tobacco smoke, and alcohol consumption.

Participants^’^ weight and height measurements were performed using standard weight and height scales (SECA, Hamburg, Germany) and procedures. Body mass index (BMI) was calculated as weight in kilograms divided by the square of the height in meters. We used BMI categories based on World Health Organization recommendations, being overweight (BMI 25.00 to 29.99 kg/m^2^), obesity class I (BMI 30.00–34.99 kg/m^2^), obesity class II (BMI 35.00–39.99 kg/m^2^), and obesity class III (≥ 40.00 kg/m^2^) [16]. In the present study, information were collected about the history of diabetes mellitus, high cholesterol as well as prescription medications for hypertension, diabetes, and high cholesterol using the questionnaire. Resting brachial blood pressure (BP) was measured using a calibrated automated BP measurement device (Omron HEM-705cp) in a sitting position using the right upper arm and an appropriately sized cuff after a period of five minutes’ rest. The average of two measures was used for analysis. Hypertension (HTN) was defined as being on anti-hypertensive medication or having a systolic blood pressure ≥ 140 mmHg or a diastolic blood pressure ≥ 90 mmHg [17].

### Statistical analysis

Data were entered into Microsoft Access and then imported into Stata version 11.0 (StataCorp LP, College Station, TX) for analysis. Demographic characteristics and CVD risk factors were evaluated in association with both smoking cigarettes and/or smokeless tobacco use. Continuous and categorical variables were assessed using t-test and/or chi-square tests, respectively. Univariate and multi-variate adjusted logistic regression models were used to examine the association of different demographic and clinical parameters with cigarette smoking and/or smokeless tobacco use. Being a current cigarette smoker and/or being a current smokeless tobacco user were the outcomes of interest in our logistic regression models. Statistical significance was assessed based on a *p*-value ≤0.05 (both sided for all tests) and the corresponding 95% confidence interval (CI).

## Results

Out of 1800 eligible males, 1375 (76%) participated in our study (433 from India, 383 from Pakistan, and 559 from Bangladesh). In Table 1 we delineate the characteristics of the study population along with their association with smoking status. The mean age of participants was 34 years (± 10) with 58% being younger than 35 years of age and approximately half (53%) having higher than secondary level education. More than two thirds (69%) of the study participants had migrated from a rural/village in their home country. The majority (70%) of participants were married, and almost half (44%) had been residing in the UAE for more than 5 years. Half of migrants (52%) were living in a labor camp. The most common occupational categories were driver (23%), laborer (17%), agricultural worker (17%), and construction worker (12%). The monthly income of study participants was relatively low with almost half of them (48%) earning less than AED 1000 per month (~USD 367). Nationality and income were significantly associated with cigarette smoking, whereas age, nationality, and education were significantly associated with smokeless tobacco use.

**Table 1:** Characteristics of Male South East Asian Migrants in Al Ain, Abu Dhabi, UAE (*N* = 1375)

Characteristics	Total N (%)	Cigarette Smoking		***p***-value	Smokeless Tobacco Use		***p***-value
		No n (%)	Yes n (%)		No n (%)	Yes n (%)	
**Age (years) - mean (SD)**	34.0 (9.9)	34.1 (9.1)	33.7 (9.1)	0.53	33.7 (9.8)	35.3 (10.1)	0.06
**Age group (years)**							
18–34	764 (58)	535 (71)	221 (29)		667 (90)	71 (10)	
35–44	333 (25)	232 (71)	97 (29)		269 (86)	43 (14)	
45–54	176 (13)	133 (76)	41 (24)		146 (86)	24 (14)	
> =55	44 (4)	35 (81)	8 (19)	0.22	33 (85)	6 (15)	0.12
**Nationality**							
India	433 (31)	337 (79)	98 (21)		385 (94)	24 (6)	
Pakistan	383 (28)	294 (77)	86 (23)		322 (88)	42 (12)	
Bangladesh	559 (41)	348 (63)	202 (37)	< 0.01	455 (84)	84 (16)	< 0.01
**Education**							
< Secondary	173 (13)	117 (69)	53 (31)		127 (78)	35 (22)	
Secondary	473 (34)	326 (70)	142 (30)		395 (87)	61 (13)	
College or higher	725 (53)	532 (75)	182 (25)	0.11	636 (92)	54 (8)	< 0.01
**Marital Status**							
Single	412 (30)	299 (74)	107 (26)		357 (90)	38 (10)	
Married	963 (70)	680 (72)	270 (28)	0.43	805 (88)	112 (12)	0.18
**Home country setting**							
Rural	932 (69)	660 (72)	259 (28)		781 (88)	111 (12)	
Urban	426 (31)	305 (73)	115 (27)	0.76	368 (91)	38 (9)	0.11
**Income (AED)**							
Mean (SD)	1828 (± 2130)	1905 (± 2298)	1667 (± 1667)	0.06	1849 (± 2153)	1602 (± 1360)	0.18
Median (IQR)	1100 (800–1100)	800	1100	0.02	1200	1100	0.64
**Income (AED)**							
< 500	371 (27)	247 (68)	117 (32)		310 (87)	45 (13)	
500–999	290 (21)	199 (69)	88 (31)		240 (88)	33 (12)	
> =2000	714 (52)	533 (76)	172 (24)	0.01	612 (89)	72 (11)	0.54
**Years in United Arab Emirates**							
< =1	239 (19)	177 (76)	56 (24)		209 (91)	21 (9)	
2–5 years	470 (37)	323 (70)	140 (30)		395 (89)	50 (11)	
6–9 years	198 (16)	124 (64)	71 (36)		172 (90)	20 (10)	
> =10 years	363 (28)	277 (77)	83 (23)	0.01	301 (86)	47 (14)	0.41
**Type of accommodation**							
At a labor camp	717 (52)	508 (72)	200 (28)		607 (87)	88 (13)	
Living with Sponsor	152 (11)	114 (77)	34 (23)		131 (88)	17 (12)	
Single accommodation	153 (11)	109 (71)	44 (29)		127 (88)	18 (12)	
Shared with relatives	184 (14)	125 (69)	56 (31)		157 (95)	9 (5)	
Shared with non-relatives	168 (12)	123 (74)	43 (26)	0.55	140 (89)	18 (11)	0.13
**Occupation**							
Driver	317 (23)	223 (71)	90 (29)		253 (84)	47 (16)	
Laborer	234 (17)	159 (68)	73 (32)		201 (89)	25 (11)	
Construction worker	172 (12)	125 (74)	44 (26)		154 (92)	13 (8)	
Agriculture worker	236 (17)	160 (68)	74 (32)		190 (85)	34 (15)	
Professional office worker	95 (7)	72 (78)	20 (22)		86 (96)	4 (4)	
Salesperson	79 (6)	57 (75)	19 (25)		73 (96)	3 (4)	
Business shop keeper	60 (4)	45 (75)	15 (25)		51 (89)	7 (11)	
Hospitality worker	71 (5)	53 (75)	18 (25)		59 (89)	7 (11)	
Tailor	70 (5)	55 (80)	14 (20)	0.52	58 (87)	9 (13)	0.01

*SD* Standard deviation

*IQR* Inter quartile range

The overall prevalence of cigarette smoking was 28% (95%CI 25–30%) and overall smokeless tobacco use was 11% (95%CI 10–13%) among South Asian migrants. The prevalence of cigarettes smoking was 21% (95%CI 17–25%), 23% (95%CI 19–27%), and 37% (95%CI 33–41%) for participants from India, Pakistan, and Bangladesh, while the prevalence of smokeless tobacco use was 6% (95%CI 4–9%), 12% (95%CI 9–15%), and 16% (95%CI 13–19%) for the three nationalities, respectively. Of the total current smokeless tobacco users, the majority were chewing tobacco including naswar (using tobacco as an oral dip), or using paan masala (65%), followed by zarda (14%), betel nut (12%), mawa (7%), and Gutka (3%). Combined tobacco use was relatively low and was found to be 2, 3, and 8% for India, Pakistani, and Bangladeshi participants, respectively. The mean (±SD) age of smoking initiation in our population was 21 (±7) years of age. A total of 4% of South Asian migrants initiated smoking below the age of 10 years, while 8, 20, and 68% initiated smoking between the ages of 11–14, 15–18, and 18+ years, respectively. Migrants smoked an average of 9 (±6) cigarettes per day. A total of 44% of migrants smoked fewer than 6 cigarettes per day, while 32% of migrants reported smoking between 6 and 10 cigarettes and 24% smoked 11 or more cigarettes per day.

In Table 2, we present different CVD risk factors in association with cigarette smoking and smokeless tobacco use. All measures of blood pressure and/or hypertension were significantly associated with cigarette smoking. However, using smokeless tobacco was not significantly associated with any CVD risk factor in this relatively young migrant population with the exception of alcohol consumption, which was significantly associated with both cigarette smoking and smokeless tobacco use. In addition, we explored the combined clustering of CVD risk factors in association with cigarette smoking and smokeless tobacco use and we did not find a significant pattern.

**Table 2:** Cardiovascular Disease Risk Factors by Smoking Status in Male South East Asian Migrants (N = 1375)

CVD Risk Factors	Total N (%)	Cigarette Smoking		***p***-value	Smokeless Tobacco Use		***p***-value
		No n (%)	Yes n (%)		No n (%)	Yes n (%)	
**Systolic blood pressure (mmHg)** - mean (±SD)	130.9 (±17.2)	128.2 (±15.8)	132.1 (±17.6)	0.01	131.0 (±17.3)	130.6 (±16.0)	0.78
**Diastolic blood pressure (mmHg)** - mean (±SD)	78.3 (±12.4)	77.1 (±12.0)	78.8 (±12.5)	0.02	78.1 (±12.4)	79.5 (±12.6)	0.21
**Blood Pressure**							
Normal	972 (71)	677 (71)	280 (29)	0.09	827 (89)	102 (11)	0.41
Grade 1	297 (21)	215 (73)	78 (27)		245 (88)	35 (12)	
Grade 2	80 (6)	66 (82)	14 (18)		67 (85)	12 (15)	
Grade 3	26 (2)	21 (81)	5 (19)		23 (96)	4 (4)	
**Hypertension**							
No	956 (73)	662 (70)	279 (30)	0.02	816 (89)	97 (11)	0.16
Yes	419 (27)	317 (76)	98 (24)		346 (87)	53 (13)	
**Diabetes Mellitus**							
No	1261 (92)	892 (72)	352 (28)	0.18	1071 (89)	135 (11)	0.36
Yes	114 (8)	87 (78)	25 (22)		91 (86)	15 (14)	
**High cholesterol**							
No	1257 (95)	909 (72)	348 (28)	0.66	1078 (86)	140 (14)	0.67
Yes	94 (5)	66 (70)	28 (30)		81 (86)	9 (14)	
**Body mass index (kg/m**^**2**^**)** - mean (±SD)	25 (4)	25 (4)	24 (5)	0.68	25 (5)	24 (4)	0.53
**Body Mass Index (kg/m**^**2**^**)**							
≤ 24.99	531 (39)	366 (70)	154 (30)	0.23	448 (88)	58 (12)	0.78
25–29.99	539 (39)	398 (75)	134 (25)		459 (89)	56 (11)	
≥ 30.0	302 (22)	213 (71)	88 (29)		252 (87)	36 (13)	
**Waist circumference (cm)** - mean (±SD)	89 (11)	89 (11)	88 (11)	0.29	89 (12)	89 (13)	0.91
**Abdominal obesity**							
No	503 (37)	354 (72)	140 (28)	0.74	427 (89)	53 (11)	0.73
Yes	872 (63)	625 (73)	237 (27)		735 (88)	97 (12)	
**Moderate exercise**							
No	1006 (75)	735 (74)	263 (26)	0.08	862 (89)	103 (11)	0.78
1–4 days per week	73 (5)	49 (69)	22 (31)		59 (89)	7 (11)	
≥ 5 days per week	269 (20)	177 (67)	87 (33)		226 (87)	34 (13)	
**Alcohol consumption**							
No	1236 (9.6)	906 (74.3)	313 (25.7)	< 0.01	1051 (89.3)	126 (10.7)	0.02
1–3 days per week	44 (3.2	19 (43.2)	25 (56.8)		35 (79.5)	9 (20.5)	
≥ 4 days per week	85 (6.2)	47 (55.9)	37 (44.1)		67 (81.7)	15 (18.3)	
**Cardiovascular disease risk factor cluster**							
No risk factors	697 (69.8)	494 (69.9)	203 (69.8)		605 (70.6)	76 (67.9)	
1 CVD risk factor	178 (17.8)	118 (16.7)	60 (20.6)	0.31	154 (17.0)	17 (15.2)	0.35
2 CVD risk factors	104 (10.4)	79 (11.2)	25 (8.6)		82 (9.6)	17 (15.2)	
3+ CVD risk factors	19 (1.9)	16 (2.3)	3 (1.0)		16 (1.9)	2 (1.8)	

Odds ratios and 95% confidence intervals for the association of different parameters with cigarette smoking are presented in Table 3. Migrants from Pakistan were more likely to smoke cigarettes and/or use smokeless tobacco in comparison with migrants from India. In addition, migrants from Bangladesh were 2 to 3 times more likely to smoke cigarettes and/or use smokeless tobacco compared to counterparts from India. Furthermore, migrants drinking alcohol were 3.3 times more likely to also smoke cigarettes, and 2.6 times more likely to use smokeless tobacco. Finally, participants with only secondary education were 2 times more likely to use smokeless tobacco compared to migrants with higher than secondary education even after adjusting for all the other variables in the model.

**Table 3:** Odds Ratios and 95% CI for different Characteristics of Male South East Asian Migrants in UAE in association with cigarette smoking and smokeless tobacco use (N = 1375)

Risk Factor	Multi-variable Adjusted OR (95% CI)			
	Cigarette Smoking	***p***-value	Smokeless Tobacco Use	***p***-value
Age	1.01 (1.00, 1.03)	0.10	1.02 (1.00–1.04)	0.05
Nationality				
Indian	ref		ref	
Pakistani	1.38 (0.94–2.03)	0.10	2.29 (1.26–4.16)	0.01
Bangladeshi	2.49 (1.76–3.53)	0.00	3.04 (1.74–5.32)	0.00
Education				
Secondary	1.07 (0.81–1.42)		2.00 (1.32–3.04)	
College or higher	ref	0.63	ref	0.00
Monthly income	1.00 (0.99, 1.00)	0.24	1.00 (1.00–1.00)	0.81
Hypertension				
No	ref		ref	
Yes	0.68 (0.51–0.92)	0.01	1.24 (0.82–1.86)	0.30
Diabetes Mellitus ^a^				
No	ref		ref	
Yes	0.69 (0.40–1.18)	0.17	1.13 (0.57–2.24)	0.73
High Cholesterol ^b^				
No	ref		ref	
Yes	1.50 (0.88–2.56)	0.13	0.84 (0.37–1.88)	0.66
Moderate exercise ^a^				
No	ref		ref	
Yes	1.11 (0.95–1.31)	0.19	0.97 (0.79–1.25)	0.97
Alcohol consumption ^a^				
No	ref		ref	
Yes	3.27 (2.17–4.94)	0.00	2.57 (1.46–4.52)	0.00

^a^ Based on self reports through an interviewer-administered questionnaire

^b^ Based on self-reports through an interviewer-administered questionnaire. Respondents who did not know their lipid status were categorized into the normal lipid category

## Discussion

To our knowledge, this is the first study to examine the prevalence of tobacco use amongst South Asian migrants living in the UAE; and report migrant smoking prevalence in the Gulf. We found a relatively high prevalence of cigarette smoking among Indian and Pakistani migrants and an even higher prevalence among Bangladeshi migrants. We found strong and significant associations between alcohol consumption and cigarette smoking and smokeless tobacco use in this migrant population. Furthermore, despite this population being relatively young, we found a clustering of CVD risk factors among smokers albeit not statistically significant.

A study from India based on the Global Adult Tobacco Survey (GATS) reported a prevalence of cigarette smoking of 15% and smokeless tobacco use of 28% among males aged 25–44 years [21]. Another study from India reported an overall prevalence of cigarette smoking of 14% and smokeless tobacco use of 8% [22]. Indian migrants in our study had a much higher prevalence of cigarette smoking (21%) and lower smokeless tobacco use (6%), a finding that may suggest an adverse impact of migration on cigarette smoking among Indian migrants. The GATS, a nationally representative household-based survey, reported that the prevalence of cigarette smoking among male Pakistanis was 19 and 11% for smokeless tobacco use [23]. In the same study, the average number of cigarettes smoked per day was 13, compared to 9 cigarettes per day in our study. Similarly, the prevalence of cigarette smoking among Bangladeshis was 43 and 17% for smokeless tobacco use [24]. We observed a relatively lower prevalence of smokeless tobacco use among migrants in our study compared to studies in their home countries [25]. Reasons for the lower smokeless tobacco use are not clear. We postulate that this result may be associated with barriers in accessing such products in the UAE compared to India, Pakistan, and Bangladesh.

South Asian migrants are also living in many other countries of Europe and in the United States. The United Kingdom, home to a large South Asian migrant population from India, Pakistan, and Bangladesh, has reported figures for smoking by country of origin over time. Cigarette smoking prevalence among Indians, Pakistanis, and Bangladeshis living in the UK was reported as 20, 29, and 40%, respectively. Smokeless tobacco use among the same migrant population in the UK was 4, 2, and 9%, respectively [26]. Tobacco use prevalence among South Asian migrants in the US was reported in two different parts of the country. In New Jersey, cigarette smoking among South Asian migrants was 12% and smokeless tobacco use was 3%. In the Northeast of the US, cigarette smoking was 9% and smokeless tobacco use was 1% [27]. The considerably lower levels of cigarette smoking and smokeless tobacco use among South Asian migrants in the US may reflect the social and contextual effect of living in a more stringent societal environment with respect to tobacco control efforts. It also re-emphasizes the need to disaggregate smoking prevalence by different ethnicities and immigrant status in order to inform relevant policies as well as preventive and smoking cessation programs for specific target populations [28].

The prevalence of cigarette smoking in our study showed an inverse association with age. On the contrary, a positive association with age was observed for smokeless tobacco use. The association between smoking prevalence and age is usually attributed to a “cohort effect”; namely, the higher prevalence of cigarette smoking in younger age groups may be associated with current societal trends in the country of residence (UAE) as opposed to smokeless tobacco use, which may represent a stronger habitual effect among older migrants being influenced in a more significant fashion from their country of origin. We also found an inverse association between educational level and tobacco use both for cigarette smoking and smokeless tobacco. This observation may be associated with a lack of awareness about health risks among those with lower educational attainment although this explanation has recently been questioned [29].

The average age of smoking initiation was 21 years in our study, which may suggest that migrants, especially from rural areas in their home countries, might have initiated smoking during migration at an older age, given the different age of smoking initiation seen among different ethnic groups. Other studies have also reported on the different age of smoking initiation among migrant populations [30]. Aggressive marketing of cigarettes to young people by tobacco companies may account for this observation. Furthermore, the slightly older age of smoking initiation in the migrant population of our study may suggest an adverse effect of migration itself. In addition to a high prevalence of smoking, we also found a strong and significant association of smoking and alcohol use in this population. Our finding is quite important with respect to planned efforts of smoking cessation. It appears that a more comprehensive approach to unhealthy behaviors combining messages for smoking cessation and moderate alcohol use may be more effective.

It is well known that migrants are “selected” and therefore differ from their home population [31]. Usually migrants have higher levels of overall health (the healthy migrant effect) but initially it is to be expected that they will share similar levels of biological and behavioral risk factors with their non-migrant peers. However, the levels of these risk factors will change during the migrants’ stay in their new environment through a process of “acculturation” [32]. Some risk factors may be reduced but others such as energy-dense diets, low activity levels, and tobacco and alcohol use may increase. In our study, there were positive associations between smoking prevalence and CVD risk factors particularly among those who smoke cigarettes. The clustering of CVD risk factors is of concern and requires a comprehensive public health response. Although the UAE has taken a number of steps to reduce tobacco consumption, including ratification of the Framework Convention on Tobacco Control, with a smoking ban in public places and health warnings including text and pictorial messaging on tobacco products, smoking prevalence remains high in all these nationalities. Prices remain extremely low and a further challenge is that smoking cessation services are not available through insurance for migrants in the UAE. Besides, the economic and health effects of smokeless tobacco use appear to be different from that of cigarette smoking and policymakers should consider specific interventions for smokeless tobacco use and address this issue in a combined effort along with smoking cessation and other programs. Studies have shown that cigarette smoking is associated with absenteeism from work causing a significant loss of work productivity [11]. Implementing strategies to control tobacco and helping workers to quit smoking resulted in increased work productivity in China, the European Union, and the US [12]. These strategies can be implemented in UAE where the working expatriate population including South Asians account for 80% of the total UAE population.

Strengths of this study include the large representative sample and the use of standardized questions to classify respondents as cigarette smokers or users of smokeless tobacco. There are some limitations in the estimates presented in this study. First, from the adapted WHO STEPS questionnaire used in this study, we only had estimates of current smoking and average number of cigarettes smoked during the last 24 h. Secondly, we were not able to assess the use of tobacco products prior and after migration, which would have provided useful information on the impact of migration. This could be important since a high proportion of migrants live in shared accommodation and therefore, may be influenced by peer pressure and behavior. Our study is also limited to male migrants. Because this was a cross-sectional study, we could not draw any conclusions on potential causal associations between cigarette smoking and the demographic, socio-economic, and CVD risk factor variables examined. Finally, it is possible that tobacco use based on self-reported questionnaires may be under-estimated and we did not validate self-reports with other objective measures of smoking such as cotinine levels. Studies have shown trends of underestimation when smoking is based on self-report and there have been varying sensitivity levels depending on the population studied. On average, the sensitivity was 88% and specificity was 89% [20]. Sensitivity values were consistently higher when cotinine was measured in saliva instead of other biomarkers such as urine or blood [33].

## Conclusions

Although the overall prevalence of tobacco smoking in the UAE is lower than many other countries in the Middle East, this hides important variations – especially the very high rates seen in South Asian migrants, and particularly in Bangladeshis. Concerted public health action is required to develop tobacco prevention and control strategies for this specific population group, which accounts for the largest proportion of the expatriate population. Being the first study of tobacco use amongst South Asian migrants in the Gulf, it provides important population-based data on the prevalence of tobacco use. There is an alarmingly high level of cigarette smoking and relatively large burden of smokeless tobacco use in these migrants originating from India, Pakistan, and Bangladesh. Given the large number of South Asian migrants in the UAE and Gulf countries, smoking cessation and prevention programs should be a public health priority for the Ministries of Health in the region. In addition, the strong association of cigarette smoking and smokeless tobacco use with alcohol drinking calls for concerted efforts in addressing multiple unhealthful behaviors with comprehensive preventive programs among migrants in this region.

## Supplementary information

**Additional file 1.** Our supplementary file is our study questionnaire titled “Chronic Diseases Prevention in Immigrants: putting CVD risk factors on surveillance screen” as has been cited in page 5, under the sub-heading, Measures of the manuscript.

## Data Availability

Future researchers can request the data sets used /or analysed during the current study from the Human Research Committee Administration (hrec.uaeu.ac.ae). The United Arab Emirates University Human Research Committee that does not allow the public release of raw dataset without prior consent from the study participant, so the authors are unable to share the de-identified datasets used in the current study via a public database.

## Abbreviations

UAE: United Arab Emirates
CVD: Cardiovascular disease
SLT: Smokeless tobacco
HTN: Hypertension
GATS: Global Adult Tobacco Survey
