# Supplementary material for: Cigarette smoking and smokeless tobacco use among male south Asian migrants in the United Arab Emirates: a cross-sectional study
Source: BMC Public Health. 2020 May 30;20:815. doi: 10.1186/s12889-020-08942-9 (PMC7260757; doi:10.1186/s12889-020-08942-9)
Supplement: Supplementary file 1 — Additional file 1. Our supplementary file is our study questionnaire titled “Chronic Diseases Prevention in Immigrants: putting CVD risk factors on surveillance screen” as has been cited in page 5, under the sub-heading, Measures of the manuscript. [file 12889_2020_8942_MOESM1_ESM.doc]

| Research Assistant Name: | | Coordinator Name: __________________ |
| --- | --- | --- |
| Date of interview: ____ / ______ /_______ | | Time of Interview: __________________ |
|  | | |
| Name of Participant: _____________________________________ | | |
| Current place of work: ___________________________________ | | |
| Contact number: _____________________ | | |
| Respondent’s Name: _____________________________________ | | |
|  |  | |
|  |  | |
| 1- | Respondent’s Gender: | |
|  | 1. Male [ ] 2. Female [ ] | |
| 2- | Nationality | |
|  | 1. India [ ] 2. Pakistan [ ] 3. Bangladesh [ ] | |
|  |  | |
| 3- | Age: _____ years | |

| **SECTION A** | | | | | | | | | | | |
| --- | --- | --- | --- | --- | --- | --- | --- | --- | --- | --- | --- |
|  | | | | | | | | | | | |
| **General Information** | | | | | | | | | | | |
|  | |  | |  | | |  | | |  | |
| A1- | | What is the highest class/level of education that completed? | | Illiterates | | | 1 | | |  | |
|  | |  | | Primary | | | 2 | | |  | |
|  | |  | | Middle | | | 3 | | |  | |
|  | |  | | Secondary | | | 4 | | |  | |
|  | |  | | Diploma, Graduate, Masters/MBA,PHD | | | 5 | | |  | |
|  | |  | |  | | | | | | | |
|  | |  | |  | | | | | | | |
| A2- | | What is your marital status? | | Married | | | 1 | | |  | |
|  | |  | | Divorced | | | 2 | | |  | |
|  | |  | | Widowed | | | 3 | | |  | |
|  | |  | | Do not know | | | 77 | | |  | |
|  | |  | | Refused | | | 99 | | |  | |
|  | |  | |  | | | | | |  | |
| A3- | | Are you blood related to your spouse? | | Yes | | | 1 | | |  | |
|  | |  | | No | | | 2 | | |  | |
|  | |  | |  | | |  | | |  | |
|  | |  | |  | | |  | | |  | |
| A4- | | IF YES-What’s the relationship with your spouse? | | First cousin1 | | | 1 | | |  | |
|  | |  | | Second cousin2 | | | 2 | | |  | |
|  | |  | | Other: ________________ | | | | | |  | |
|  | |  | |  | | |  | | |  | |
| A5- | | How many children do you have? | | Boys: _____ Girls: ______ | | | | | |  | |
|  | |  | |  | | | | | |  | |
| A6- | | In which type of family system do you live? | | Joint family system1 | | | 1 | | |  | |
|  | | 1. Husband , wife, parents, in-laws and other | | Nuclear2 | | | 2 | | |  | |
|  | | 2. Only husband, wife and children | |  | | | | | |  | |
|  | |  | |  | | | | | |  | |
| A7- | | What is your Mother Language? | | | _______________________ | | |  | |  | |
|  | |  | | |  | | |  | |  | |
|  |  | |  | | |  | | |  | |  |
| A8- | Are you currently employed or engaged in any business? | | Yes | | | 1 | | |  | |  |
|  | If Yes | | No | | | 2 | | |  | |  |
|  |  | |  | | |  | | |  | |  |
| A9- | What Nature of your work/employment? | | _____________________ | | | | | |  | |  |
|  |  | |  | | | | | |  | |  |
| A10- | How Many Years in the current work? | | _______ | | |  | | |  | |  |
|  |  | |  | | |  | | |  | |  |
| A11- | Monthly salary/income? (Dh) | |  | | |  | | |  | |  |
|  |  | |  | | |  | | |  | |  |
|  | If Not employed | |  | | |  | | |  | |  |
|  |  | |  | | |  | | |  | |  |
| A12- | Reason for not being employed. | | Student | | | 1 | | |  | |  |
|  | *(Multiple responses are possible)* | | Housewife | | | 2 | | |  | |  |
|  |  | | Retired | | | 3 | | |  | |  |
|  |  | | Jobless | | | 4 | | |  | |  |
|  |  | | Other | | | 5 | | |  | |  |
|  |  | | Specify _____________ | | | | | |  | |  |

| **SECTION B** | | | | |
| --- | --- | --- | --- | --- |
|  | | | | |
| In this section I would like to ask you about your health | | | | |
|  |  |  |  |  |
| B1- | What would you say about your health in general? | Excellent | 1 |  |
|  |  | Very Good | 2 |  |
|  |  | Good | 3 |  |
|  |  | Fair | 4 |  |
|  |  | Poor | 5 |  |
|  |  |  |  |  |
| B2- | How much you are satisfied with your life? | Very happy | 1 |  |
|  |  | Happy | 2 |  |
|  |  | Fair | 3 |  |
|  |  | Not happy | 4 |  |
|  |  | Very unhappy | 5 |  |
|  |  |  |  |  |
|  |  |  |  |  |
| B3- | Before this interview, has your blood pressure ever been checked? | Yes | 1 |  |
|  |  | No | 2 |  |
|  |  |  |  |  |
| B4- | Has a health care provider ever told you that you have high blood pressure also called hypertension? | Yes | 1 |  |
|  |  | No | 2 |  |
|  |  |  |  |  |
|  | *If ‘No’ move to Question B10* |  |  |  |
|  |  |  |  |  |
| B5- | What was your age when you were told that you have high blood pressure? | ______ years | |  |
|  |  |  |  |  |
| B6- | Because of your high blood pressure have you were ever being told by a health care provider to take prescribed medicine? | Yes | 1 |  |
|  |  | No | 2 |  |
|  |  |  |  |  |
| B7- | Are you currently (since last month) taking any medicine for blood pressure? | Yes | 1 |  |
|  |  | No | 2 |  |

| B8- | Please, mention the names and dosages of medicines you are using. If you do not know the names of medicines, then kindly show me these medicines. | | | |
| --- | --- | --- | --- | --- |
| No. | **Name of Medicines** | **Type**  1. Tablet, 2. Capsules, 3. Syrupp/Susp, 4. Injection, 5. Inhalers, 6. Others |  | |
|  |  |  | **Dosage** | **per day** |
| 01 |  |  |  |  |
| 02 |  |  |  |  |
| 03 |  |  |  |  |
| 04 |  |  |  |  |
| 05 |  |  |  |  |

| B9- | Have you taken your blood pressure medicine today or during the last 2 days? | Yes today | 1 |  |
| --- | --- | --- | --- | --- |
|  |  | Yes in last two days | 2 |  |
|  |  | No | 3 |  |
|  | If not, what was the reason for not taking the blood pressure medicine? | _________________________ |  |  |
|  |  |  |  |  |
|  |  |  |  |  |
|  |  |  |  |  |
| B10- | Did any of your first degree relatives (mother, father, brother, sister, son, daughter) were told by a doctor or other health professional that they had high blood pressure? | Yes | 1 |  |
|  |  | No | 2 |  |
|  |  | Don’t know | 3 |  |
|  |  |  |  |  |
| **Heart Disease** | | | | |
| B11- | Has a doctor or other health professional ever told you that you have Heart Disease? | Yes | 1 |  |
|  |  | No | 2 |  |
|  |  |  |  |  |
|  | *If Not – Move to Question B16* |  |  |  |
|  |  |  |  |  |
| B12- | What was that heart disease? | Angina | 1 |  |
|  |  | Heart Attack | 2 |  |
|  |  | Other (specify)_____________ | 3 |  |
|  |  |  | |  |
|  |  |  |  |  |
| B13- | How old were you when you were told? | _________years | |  |
|  |  |  |  |  |
| B14- | Were you prescribed treatment for it? | Yes | 1 |  |
|  |  | No | 2 |  |
|  | *If Not – Move to Question B16* |  |  |  |
|  |  |  |  |  |
| B15- | If yes, the type of treatment was? | Medical treatment only | 1 |  |
|  |  | Medical & surgical treatment | 2 |  |
|  |  | Other (specify) _____________ | 3 |  |
|  |  |  |  |  |
| B16- | Did any of your first-degree relatives (mother, father, brother, sister, son, daughter) were told by a doctor or other health professional that they had a heart attack before the age of 55 years? | Yes | 1 |  |
|  |  | No | 2 |  |
|  |  | Don’t Know | 3 |  |
|  |  |  |  |  |
|  | **Cholesterol and Triglyceride** | |  |  |
|  |  |  |  |  |
| B17- | Have you ever had your blood lipids measured (Cholesterol or | Yes | 1 |  |
|  | triglycerides)? | No | 2 |  |
|  |  |  |  |  |
| B18- | Have you ever been told by a doctor, or another health | Yes | 1 |  |
|  | professional that your blood cholesterol level was high? | No | 2 |  |
|  |  |  |  |  |
|  | *If NO – Move to Question B20* |  |  |  |
|  |  |  |  |  |
| B19- | Are you currently taking any treatment for your high | Yes | 1 |  |
|  | cholesterol? | No | 2 |  |
|  |  |  |  |  |
|  | **Kidney Disease** | |  |  |
|  |  |  |  |  |
| B20- | Has a doctor or other health professional ever told you that | Yes | 1 |  |
|  | you have a kidney disease? | No | 2 |  |
|  |  |  |  |  |
| B21 | If yes, what kind of kidney disease you had? | _________________________ |  |  |
|  |  |  |  |  |
|  |  |  |  |  |
|  | **Diabetes** | |  |  |
|  |  |  |  |  |
| B22- | Has a health care provider ever told you that you have | Yes | 1 |  |
|  | Diabetes? | No | 2 |  |
|  |  |  |  |  |
|  | *If not - Move to Question B29* |  |  |  |
|  |  |  |  |  |
| B23- | How was your diabetes diagnosed? | Had symptoms | 1 |  |
|  | *(More than one response is possible)* | Screening test (high blood sugar) | 2 |  |
|  |  | Sugar in urine | 3 |  |
|  |  | Other (Specify) ____________ | 4 |  |
|  |  |  |  |  |
| B24- | For how long you have been told that you have diabetes? | ____________ years |  |  |
|  |  |  |  |  |
|  | *In case less that 1 year, mark ‘0’* |  |  |  |
|  |  |  |  |  |
| B25- | Were you prescribed any treatment or restricted diet for your | Yes | 1 |  |
|  | diabetes? | No | 2 |  |
|  |  |  |  |  |
| B26- | **If yes**, Type of current treatment you are taking for diabetes? | Insulin | 1 |  |
|  | *(More than one response is possible)* | Tablets | 2 |  |
|  |  | Restricted diet | 3 |  |
|  |  | Other (Specify) ____________ | 4 |  |
|  |  |  |  |  |
| B27- | Have you taken your diabetes medicine today or during the | Yes, today | 1 |  |
|  | last 2 days? | Yes, in the last 2 days | 2 |  |
|  |  | No, medication was taken | 3 |  |
|  |  | Why? ____________________ |  |  |
|  |  |  |  |  |
| B28 | Do you have a blood sugar checking device at your home? | Yes | 1 |  |
|  |  | No | 2 |  |
|  |  | I do not know | 3 |  |
|  |  |  |  |  |
|  |  |  |  |  |
|  |  |  |  |  |
| B29- | Did any of your first-degree relatives (mother, father, brother, | Yes | 1 |  |
|  | sister, son, daughter) were told by a doctor or other health | No | 2 |  |
|  | professional that they had diabetes? | I do not know | 3 |  |
|  |  |  |  |  |
|  | **Stroke** | |  |  |
|  |  |  |  |  |
| B30- | Has a doctor or other health professional ever told you that | Yes | 1 |  |
|  | you had a stroke? | No | 2 |  |
|  |  | I do not know | 3 |  |
|  | If No – Move to Question B33 |  |  |  |
|  |  |  |  |  |
| B31- | Do you know the cause of stroke? | Yes | 1 |  |
|  |  | No | 2 |  |
|  |  |  |  |  |
| B32- | If Yes, what is the major cause? Specify: | _________________________ |  |  |
|  |  |  |  |  |
| B33- | Did any of your first degree relatives (mother, father, brother, | Yes | 1 |  |
|  | sister, son, daughter) were told by a doctor or other health | No | 2 |  |
|  | professional that they had a stroke? |  |  |  |
|  |  |  |  |  |
|  | **Injury** | |  |  |
|  |  |  |  |  |
| B34- | Did you get an injury in the past 12 month that needed | Yes | 1 |  |
|  | treatment? | No | 2 |  |
|  |  |  |  |  |
|  | *If No – Move to Question B39* |  |  |  |
|  |  |  |  |  |
| B35- | How did the injury occur? | _________________________ |  |  |
|  |  |  |  |  |
| B36- | Where the injury occurred? | _________________________ |  |  |
|  |  |  |  |  |
| B37- | What was the cause of that injury? | _________________________ |  |  |
|  |  |  |  |  |
| B38- | Where was the treatment received? | Govt. hospital | 1 |  |
|  |  | Primary H. Care | 2 |  |
|  |  | Private Hospital | 3 |  |
|  |  | Other (specify) _____________ | 4 |  |
|  |  |  |  |  |
|  | **Mother and Father Health** | |  |  |
|  |  |  |  |  |
| B39- | How is the health of your father? | Good | 1 |  |
|  |  | Sick (specify) ______________ | 2 |  |
|  |  | Died (Reason) _____________ | 3 |  |
|  |  |  |  |  |
| B40- | How is the health of your mother? | Good | 1 |  |
|  |  | Sick (specify) ______________ | 2 |  |
|  |  | Died (Reason) _____________ | 3 |  |
|  |  |  |  |  |

| **SECTION C** | | | | |
| --- | --- | --- | --- | --- |
|  |  |  |  |  |
| **International Physical Activity Questionnaire** | | | | |
| We are interested in finding out about the kinds of physical activities that people do as part of their everyday lives. The questions will ask you about the time you spent being physically active in the **last 7 days**. Please answer each question even if you do not consider yourself to be an active person. Please think about the activities you do at work, as part of your house and yard work, to get from place to place, and in your spare time for recreation, exercise or sport. | | | | |
|  |  |  |  |  |
|  | **Vigorous Physical Activity** | |  |  |
|  | Think about all the **vigorous** activities that you did in the **last 7 days**. Vigorous physical activities refer to activities that take hard physical effort and make you breathe much harder than normal. Think *only* about those physical activities that you did for at least 10 minutes at a time. | |  |  |
|  |  |  |  |  |
| C1- | During the **last 7 days**, on how many days did you do | None | 1 |  |
|  | **vigorous** physical activities like heavy lifting, digging, | 1 Day | 2 |  |
|  | aerobics or fast bicycling**?** | 2 Days | 3 |  |
|  |  | 3 Days | 4 |  |
|  |  | 4 Days | 5 |  |
|  |  | 5 Days | 6 |  |
|  |  | 6 Days | 7 |  |
|  |  | 7 Days | 8 |  |
|  |  | Do not Know | 77 |  |
|  |  | Refuse | 99 |  |
|  |  |  |  |  |
|  | *If ‘None’, ‘Do not know’, ‘Refuse’ – Skip to Question C3* |  |  |  |
|  |  |  |  |  |
| C2- | How much time did you usually spend doing **vigorous** | _____ hours/day |  |  |
|  | physical activities on one of those days? | _____ minutes/day |  |  |
|  |  |  |  |  |
|  |  |  |  |  |
|  |  |  |  |  |
|  |  |  |  |  |
|  | **Moderate Physical Activity** | |  |  |
|  | Think about all the **moderate** activities that you did in the **last 7 days**. **Moderate** activities refer to activities that take moderate physical effort and make you breathe somewhat harder than normal. Think only about those physical activities that you did for at least 10 minutes at a time. | |  |  |
|  |  | |  |  |
| C3- | During the **last 7 days**, on how many days did you do | None | 1 |  |
|  | **moderate** physical activities like carrying light loads, | 1 Day | 2 |  |
|  | bicycling at a regular pace, or doubles tennis? | 2 Days | 3 |  |
|  | Do not include walking. | 3 Days | 4 |  |
|  |  | 4 Days | 5 |  |
|  |  | 5 Days | 6 |  |
|  |  | 6 Days | 7 |  |
|  |  | 7 Days | 8 |  |
|  |  | Do not Know | 77 |  |
|  |  | Refuse | 99 |  |
|  |  |  |  |  |
|  | *If ‘None’, ‘Do not Know’, ‘Refuse’ – Skip to Question C5* |  |  |  |
|  |  |  |  |  |
| C4 | How much time did you usually spend doing **moderate** | _____ hours/day |  |  |
|  | physical activities on one of those days? | _____ minutes/day |  |  |
|  |  |  |  |  |
|  | **Walking** | |  |  |
|  | Think about the time you spent walking in the **last 7 days**. This includes at work and at home, walking to travel from place to place, and any other walking that you might do solely for recreation, sport, exercise, or leisure. | |  |  |
|  |  |  |  |  |
| C5- | During the **last 7 days**, on how many days did you **walk** for at | None | 1 |  |
|  | least 10 minutes at a time? | 1 Day | 2 |  |
|  |  | 2 Days | 3 |  |
|  |  | 3 Days | 4 |  |
|  |  | 4 Days | 5 |  |
|  |  | 5 Days | 6 |  |
|  |  | 6 Days | 7 |  |
|  |  | 7 Days | 8 |  |
|  |  | Do not Know | 77 |  |
|  |  | Refuse | 99 |  |
|  |  |  |  |  |
|  | *If ‘None’, ‘Do not Know’, ‘Refuse’ – Skip to Question C7* |  |  |  |
|  |  |  |  |  |
| C6- | How much time did you usually spend **walking** on one of | _____ hours/day |  |  |
|  | those days? | _____ minutes/day |  |  |
|  |  |  |  |  |
| C7- | During the **last 7 days,** how much time did you spent sitting | _____ hours/day |  |  |
|  | on weekdays during the **last 7 days**? Include time spent at work, at home, while doing course work and during leisure time. This may include time spent sitting at a desk, visiting friends, reading, or sitting or lying down to watch television. | _____ minutes/day |  |  |
|  |  |  |  |  |
|  | **Sitting Time** | |  |  |
|  |  |  |  |  |
| C8- | During the **last 7 days**, on how many days did you spent | None | 1 |  |
|  | sitting or lying down to watch television, work / play | 1 Day | 2 |  |
|  | computer, play electronic games? | 2 Days | 3 |  |
|  |  | 3 Days | 4 |  |
|  |  | 4 Days | 5 |  |
|  |  | 5 Days | 6 |  |
|  |  | 6 Days | 7 |  |
|  |  | 7 Days | 8 |  |
|  |  | Do not Know | 77 |  |
|  |  | Refuse | 99 |  |
|  |  |  |  |  |
|  | *If ‘None’, ‘Do not Know’, ‘Refuse’ – Skip to Question D1* |  |  |  |
|  |  |  |  |  |
| C9- | How much time did you usually spend **sitting** on one of those | _____ hours/day |  |  |
|  | days? | _____ minutes/day |  |  |
|  |  |  |  |  |

| **SECTION D** | | | | |
| --- | --- | --- | --- | --- |
|  |  |  |  |  |
| **Lifestyle** | | | | |
| D1- | Have you ever smoked cigarettes/cigars/biddies/shisha? | Never | 1 |  |
|  |  | Former smoker | 2 |  |
|  |  | Current smoker | 3 |  |
|  |  |  |  |  |
|  | *If ‘Never smoked’ – Move to Question D14* |  |  |  |
|  |  |  |  |  |
|  | **Former Smoker** | |  |  |
| D2- | At what age did you start smoking? | ________ Years |  |  |
|  |  |  |  |  |
| D3- | For how many years have you /did you smoked? | ________ Years |  |  |
|  |  |  |  |  |
| D4- | When did you quit smoking? | ________ Years |  |  |
|  |  | ________ Years |  |  |
|  |  |  |  |  |
| D5- | What was the average number of cigarettes per day that you?  smoked in the past? | _____ cigarettes |  |  |
|  |  |  |  |  |
|  | **Current Smoker** |  |  |  |
| D6- | At what age did you start smoking? | ________ Years |  |  |
|  |  |  |  |  |
| D7- | For how many years have you /did you smoked? | ________ Years |  |  |
|  |  |  |  |  |
| D8- | When did you quit smoking? | ________ Years |  |  |
|  |  | ________ Years |  |  |
|  |  |  |  |  |
| D9- | What was the average number of cigarettes per day that you  smoked in the past? | _____ cigarettes |  |  |

|  | During the last week, how many times did you use cigarettes/ cigars/ water/pipe or shisha/naswar? | D10-Cigarettes | D11- Cigar/shisha | D12-  Paan masla/zarda/betelnut/  mava/Gutka | D13-  Naswar/chewed tobacco |
| --- | --- | --- | --- | --- | --- |
| 1 | None |  |  |  |  |
| 2 | Once /week |  |  |  |  |
| 3 | 2-3 times /week |  |  |  |  |
| 4 | 4-5 times/ week |  |  |  |  |
| 5 | Every day |  |  |  |  |

| D14- | On how many of the past 7 days did someone smoke in your | None | 1 |  |
| --- | --- | --- | --- | --- |
|  | indoor workplace while you were there? | Once / week | 2 |  |
|  |  | 2-3 times / week | 3 |  |
|  |  | 4-5 times / week | 4 |  |
|  |  | Every day | 5 |  |
|  |  | Do not know | 77 |  |
|  |  |  |  |  |
| D15- | On how many of the past 7 days did any one smoke in your | None | 1 |  |
|  | home while you were there? | Once / week | 2 |  |
|  |  | 2-3 times / week | 3 |  |
|  |  | 4-5 times / week | 4 |  |
|  |  | Every day | 5 |  |
|  |  | Do not know | 77 |  |
|  |  |  |  |  |
| D16- | How often are cigarettes smoked inside your home in a typical | Never | 1 |  |
|  | week? | Once in a week | 2 |  |
|  |  | 2 – 3 times/week | 3 |  |
|  |  | 4 – 5 times/week | 4 |  |
|  |  | Daily | 5 |  |
|  |  | Do not know | 77 |  |

| **SECTION E** | | | | |
| --- | --- | --- | --- | --- |
|  |  |  |  |  |
| **Questions related to diet at home** | | | | |
|  |  |  |  |  |
| E1- | Do you add extra salt on your food? | Yes | 1 |  |
|  |  | No | 2 |  |
|  | *If No – Skip to SECTION F* |  |  |  |
|  |  |  |  |  |
| E2- | How often do you add salt on your food? | Occasionally (< 7 / week) | 1 |  |
|  |  | Often (> 7 / week) | 2 |  |
|  |  | Always | 3 |  |
|  |  |  |  |  |
|  |  |  |  |  |
| **SECTION F** | | | | |
|  |  |  |  |  |
| **Physical Examination** | | | | |
|  |  |  |  |  |
| F1- | Have you smoked a cigarette or taken coffee or tea in the last | Yes | 1 |  |
|  | 30 minutes? | No | 2 |  |
|  |  |  |  |  |
| F2- | Systolic Blood Pressure |  |  |  |
|  | Reading 1 | _____ mmHg |  |  |
|  | Reading 2 (5 minutes after reading 1) | _____ mmHg |  |  |
|  | Reading 2 (5 minutes after reading 2) | _____ mmHg |  |  |
|  |  |  |  |  |
| F3- | Diastolic Blood Pressure |  |  |  |
|  | Reading 1 | _____ mmHg |  |  |
|  | Reading 2 (5 minutes after reading 1) | _____ mmHg |  |  |
|  | Reading 2 (5 minutes after reading 2) | _____ mmHg |  |  |
|  |  |  |  |  |
| F4- | Pulse |  |  |  |
|  | Reading 1 | _____ bpm |  |  |
|  | Reading 2 (5 minutes after reading 1) | _____ bpm |  |  |
|  | Reading 2 (5 minutes after reading 2) | _____ bpm |  |  |
|  | **Anthropometric measurements** | |  |  |
| F5- | Height |  |  |  |
|  | Reading 1 | _____ cm |  |  |
|  | Reading 2 | _____ cm |  |  |
|  |  |  |  |  |
| F6- | Weight |  |  |  |
|  | Reading 1 | _____ kg |  |  |
|  | Reading 2 | _____ kg |  |  |
|  |  |  |  |  |
| F7- | Waist circumference |  |  |  |
|  | Reading 1 | _____ cm |  |  |
|  | Reading 2 | _____ cm |  |  |
|  |  |  |  |  |
| F8- | Hip circumference |  |  |  |
|  | Reading 1 | _____ cm |  |  |
|  | Reading 2 | _____ cm |  |  |
|  |  |  |  |  |
|  |  |  |  |  |
| **SECTION G** | | | | |
|  |  |  |  |  |
| **Any laboratory test results** | | | | |
|  |  |  |  |  |
| G1- | Fasting glucose | ________ (units) |  |  |
|  |  |  |  |  |
| G2- | HbA1c | ________ (units) |  |  |
|  |  |  |  |  |
| G3- | Total Cholesterol | ________ (units) |  |  |
|  |  |  |  |  |
| G4- | LDL | ________ (units) |  |  |
|  |  |  |  |  |
| G5- | HDL | ________ (units) |  |  |
|  |  |  |  |  |
| G6- | Triglycerides | ________ (units) |  |  |
